# Selective Depletion of Cancer Cells with Extrachromosomal DNA via Lentiviral Infection

**DOI:** 10.1158/2767-9764.CRC-25-0144

**Published:** 2025-08-28

**Authors:** Eunhee Yi, Amit D. Gujar, Dacheng Zhao, Kentaro Suina, Xue Jin, Katharina Pardon, Qinghao Yu, Larisa Kagermazova, Emmanuel E. Korsah, Noah A. Dusseau, Jef D. Boeke, Anton G. Henssen, Roel G.W. Verhaak

**Affiliations:** 1 Department of Physiology, College of Human Medicine, Michigan State University, East Lansing, Michigan.; 2 Department of Neurosurgery, School of Medicine, Yale University, New Haven, Connecticut.; 3 University of Connecticut Health Center, Farmington, Connecticut.; 4 Department of Pediatric Oncology/Hematology, Charité-Universitätsmedizin Berlin, Berlin, Germany.; 5 Experimental and Clinical Research Center (ECRC) of the MDC and Charité Berlin, Berlin, Germany.; 6 Max-Delbrück-Centrum für Molekulare Medizin (BIMSB/BIH), Berlin, Germany.; 7 Department of Biochemistry and Molecular Pharmacology, Institute for Systems Genetics, NYU Langone Health, New York, New York.; 8 Department of Biomedical Engineering, NYU Tandon School of Engineering, Brooklyn, New York.; 9 Department of Physiology, College of Natural Science, Michigan State University, East Lansing, Michigan.; 10 Berlin Institute of Health, Berlin, Germany.; 11 German Cancer Consortium (DKTK), partner site Berlin and German Cancer Research Center (DKFZ), Heidelberg, Germany.; 12 Department of Neurosurgery, Amsterdam University Medical Centers/VUmc, Amsterdam, the Netherlands.

## Abstract

Extrachromosomal DNA (ecDNA), a major focal oncogene amplification mode found across cancer, has recently regained attention as an emerging cancer hallmark, with a pervasive presence across cancers. With technical advancements such as high-coverage sequencing and live-cell genome imaging, we can now investigate the behaviors and functions of ecDNA. However, we still lack an understanding of how to eliminate ecDNA. We observed depletion of cells containing ecDNA during lentiviral but not transposon-based transduction, whereas we sought to investigate the mechanism of ecDNA behavior. This discovery may provide critical information on utilizing a lentiviral system in emerging ecDNA research. Additionally, this observation suggests specific sensitivities for cells with ecDNA.

**Significance:**

ecDNA is an essential factor in cancer progression. We found that a group of cancer cells with ecDNA is selectively depleted after lentiviral infection. This finding provides promise for ecDNA-specific targeting, suggests the need for caution in using lentivirus, and offers alternative ways to study ecDNA.

## Introduction

Extrachromosomal DNA (ecDNA) is a double-stranded circular DNA that is physically separated from chromosomes and contains multiple areas highly accessible to transcriptional machinery (1). This circular DNA often amplifies major oncogenes such as EGFR, MYC, and CDK4, and patients with cancer who have oncogene amplification on ecDNA show poorer survival than those with the same oncogene amplified on chromosomes (2). ecDNAs lack centromeres and lead to unequal segregation of their copies during cell division, resulting in substantial intratumoral heterogeneity (3). Although ecDNA was first discovered in the 1960s (4), it has gained attention recently as we now have a better understanding of ecDNA’s function and its clinical significance (2, 3, 5–7). As ecDNA is an emerging area in cancer research, many studies focusing on its biological functions and mechanisms are expected to be released over the next several years.

Introducing plasmid DNA into eukaryotic cells is a common method to study genes or deliver nucleic acids that can produce reporter modules such as fluorescent proteins (8). Lentiviral transduction is an efficient way to transfer genetic material to the target cells and has the natural ability to integrate foreign DNA into the genomes of host cells. Therefore, the transduced cells can express foreign genes stably. This lentiviral transduction has been actively used for CRISPR-Cas9 genetic screens in mammalian cells to identify essential molecular mechanisms for various biological processes by knocking out specific genes (9, 10).

We serendipitously discovered that lentiviral transduction, followed by antibiotic selection, dramatically reduced the fraction of cancer cells containing ecDNA. In this study, we identified the cause of this specific loss of cells with ecDNA and suggested alternative ways to achieve transgene delivery to cells without interfering with ecDNA status.

## Materials and Methods

### Cell cultures and cell lines

The human prostate cancer cell line acquired from a 62-year-old White male, PC3, was a gift from Dr. Paul Mischel at Stanford University and was cultured in F12-K (ATCC, 30-2004) with 10% FBS (VWR, 97068-085). The parental HeLa-S3 cell line (ATCC, CCL-2.2; RRID:CVCL_0058), acquired from a 31-year-old Black female, was purchased from ATCC and cultured in DMEM (Gibco, 11054020) supplemented with 10% FBS (VWR, 97068-085), 1% penicillin and streptomycin (Gibco, 15140122), and 2 mmol/L L-glutamine (Gibco, 25030081). The COLO320DM (ATCC, CCL-220; RRID:CVCL_0219) and COLO320HSR (ATCC, CCL-220.1; RRID:CVCL_0220) cell lines, acquired from a 55-year-old White female were purchased from ATCC and cultured in RPMI 1640 (ATCC, 30-2001) supplemented with 10% FBS (VWR, 97068-085). All cell lines were not authenticated and were routinely tested for *Mycoplasma* contamination using the MycoAlert Mycoplasma Detection Kit (Lonza).

### Generation of the methotrexate-resistant HeLa single-cell clones

Parental HeLa S3 cells were treated with 160 nmol/L methotrexate for 2 to 6 weeks. Resistant cells were harvested and subjected to single-cell cloning. The clones were allowed to expand in low methotrexate concentrations to obtain clonal lines, which were screened for dihydrofolate reductase (*DHFR*) copy number. Clones with the highest copy number were treated with progressively increasing methotrexate concentrations and characterized by DAPI staining and FISH analyses of metaphase spreads to identify clones with and without ecDNAs.

### Generation of PC3 single-cell clones

Single PC3 cells were plated in each well of a 96-well plate using a FACSAria cell sorter. Each single cell was then cultured until a colony was visible. Individual colonies from each well were subsequently passed into bigger plates and expanded. The *MYC* amplification status of each clone was validated by FISH analysis.

### General lentivirus production and transduction

The 293T cells (ATCC, CRL-3216; RRID:CVCL_0063) were seeded onto poly-L-lysine–coated plates to obtain 70% to 80% confluence on the following day when the cells were transfected with packaging and envelope plasmids along with the lentiviral plasmid expressing specific genes using Lipofectamine 3000 transfection reagent. The medium was changed 6 hours after transfection. On the next day (29 hours after transfection), lentiviral supernatants were collected and mixed with Lenti-X Concentrator as per the manufacturer’s instructions. Lentiviral supernatants with the concentrator were incubated overnight at 4°C. Lentiviruses were then pelleted using centrifugation, and the pellets were resuspended in PBS; aliquots were prepared and stored at −80°C until use.

For transduction, cells were seeded in 24- or 6-well plates to obtain 60% to 70% confluence on the following day when they were transduced with the indicated lentiviruses using 8 μg/mL polybrene or transfected with PiggyBac system plasmids using Lipofectamine 3000 transfection reagent. Treatment with a selection marker was started 24 or 48 hours after transfection/transduction. Medium with selection was changed every 2 to 3 days. Cells were harvested 1 or 2 weeks after starting selection and processed for metaphase spread preparation and FISH analysis.

For the scrambled lentivirus experiment, we used the LentiArray CRISPR nontargeting control lentivirus particle (Invitrogen, #A32062).

### Transduction efficiency test for 72-hour infection

293T cells were seeded onto cell culture–treated 10 cm plates to obtain 70% to 80% confluence on the following day when the cells were transfected with packaging and envelope plasmids along with the lentiviral plasmid expressing GFP (pHAGE-EF1α-EGFP-Puro, 9 kb) using Lipofectamine 2000 transfection reagent. Forty-eight hours later, lentiviral supernatants were collected, filtered (0.45 μmol/L), aliquoted, and stored at −80°C until use.

For transduction, PC3 clones were seeded in six-well plates to obtain 60% to 70% confluence on the following day (0.1 × 10^6^ cells/well). The following day, the cells were transduced with equal amounts of lentivirus. Twenty-four hours after infection, the lentiviral media were replenished with fresh media. To assess the infection efficiency, GFP-expressing cells were quantified via flow cytometry 72 hours after infection.

Cells were washed twice with PBS and resuspended in FACS buffer (PBS with 2% FBS). To ensure a single-cell suspension, samples were passed through cell strainer tubes (Fisher Scientific, #08-771-23) before analysis. Flow cytometry was performed using a Sony SH800 cell sorter, collecting 100,000 events per sample. GFP fluorescence was detected using the FITC channel (488 nm excitation). Data were analyzed using FlowJo, with GFP-positive cell percentages quantified after gating on live, single-cell populations.

### FISH analysis

PC3 cells with and without lentiviral transduction were treated with 80 ng/mL Colcemid (Roche, 10-295-892-001) for 5 hours (24 hours for HeLa-MTX-Res cells) and harvested to prepare metaphase spreads using standard cytogenetic procedures. Metaphase spreads were subjected to FISH analysis using probes binding to *MYC* and chromosome 8 (*DHFR* and chromosome 5 for HeLa-MTX-Res cells). A hybridization buffer (Empire Genomics) mixed with probes (Empire Genomics) was applied to the slides, and the slides were denatured at 75°C for 5 minutes. The slides were then immediately transferred and incubated at 37°C overnight. The posthybridization wash was with prewarmed 0.4× saline sodium citrate at 75°C for 1 minute, followed by a second wash with 2× saline sodium citrate/0.05% Tween 20 for 2 minutes at room temperature. The slides were then briefly rinsed with water and air-dried. The VECTASHIELD mounting medium with DAPI (Vector Laboratories) was applied, and the coverslip was mounted onto a glass slide. Tissue images were scanned under the Leica STED 3×/DLS Confocal (RRID:SCR_024405) or the Leica Stellaris 5 Confocal (RRID:SCR_024663) with an oil-immersion objective (40×). As excitation lasers, 405, 488, and 561 nm were used. A Z-stack acquired at a 0.3 to 0.5 μm step size was performed, and all analysis was conducted based on maximum intensity projection images of the 3D volume of the cells. Images were acquired and processed by LAS X software (RRID:SCR_013673). To minimize bias in FISH imaging, we randomly took at least 30 images of metaphase cells in the order in which the cells were shown on the slide.

### Real-time qPCR

The total RNA was extracted from ecDNA-positive (ecDNA+) and homogenously staining region-positive (HSR+) clones using an RNeasy kit (QIAGEN) following the manufacturer’s instructions. The RNA quality and concentration were assessed using NanoDrop spectrophotometers (Thermo Fisher Scientific; RRID:SCR_023005). cDNA was synthesized using a High-Capacity cDNA Reverse Transcription kit (Applied Biosystems) according to the manufacturer’s protocol; then, a master mix containing SYBR Green PCR Master Mix (Applied Biosystems), forward and reverse primers, and cDNA template was prepared. For the real-time PCR cycling conditions, the initial denaturation was at 95°C for 2 minutes; the denaturation was at 95°C for 15 seconds; the annealing/extension was at 57°C for 30 seconds and at 72°C for 1 minute (repeat for 40 cycles); and the melting curve analysis was performed at 95°C for 15 seconds, at 60°C for 1 minute, and at 95°C for 15 seconds. Data analysis was performed using QuantStudio Design & Analysis Software (Applied Biosystems; RRID:SCR_018712). Ct values were normalized to a reference gene (GAPDH), and relative expression was calculated using the ΔΔCt method.

### Cell viability assay

Cell viability was tested using CellTiter-Glo (Promega) according to the manufacturer’s protocol. Luminescence was measured using the Tecan Infinite 200 PRO microplate reader (Tecan Life Sciences; RRID:SCR_020543).

### Flow cytometry assay

For PC3-derived cells and COLO320 cells, the cells were transduced with lentivirus carrying GFP for 24 hours. For neuroblastoma cell lines, the cells were transduced with lentivirus carrying GFP for 48 hours. The transduced cells were harvested, rinsed with DPBS without calcium and magnesium, and resuspended in FACS buffer (0.1% BSA in DPBS). The number of GFP-positive cells was analyzed using FACSAria II (RRID:SCR_018934). For the cell death analysis, transduced cells were stained with cell death markers, 7-aminoactinomycin D or propidium iodide, using the manufacturer’s protocols and subjected to flow cytometry analysis.

### Whole-genome sequencing and data processing

Genomic DNA was isolated using DNeasy Blood & Tissue Kits (QIAGEN). The genomic DNA samples after whole-genome sequencing (WGS) library preparation were then sequenced on the Illumina NovaSeq (RRID:SCR_016387) at 10 to 30× coverage. Raw reads in fastq files were aligned to hg19 using BWA-MEM version 0.7.19 (arXiv.1307.3997). The duplicated reads were marked by the MarkDuplicates tool from the GATK Best Practices pipeline version 4.5.0.0 (arXiv.1307.3997; ref. 11). Copy-number ratios in 1 kb windows were computed using GATK’s CollectFragmentCounts and then segmented with ModelSegment according to GATK’s Best Practice pipeline. The *MYC* amplicons were identified with AmpliconArchitect (12). The aligned BAM files were processed with AmpliconSuite (version 1.3.8; bioRxiv.2024.05.06.592768) to identify ecDNA structure with default settings using CNVkit to identify the seeding region.

### Statistical analysis

All sample sizes and statistical methods are indicated in the corresponding figures or figure legends. All statistical tests were performed in GraphPad Prism (RRID:SCR_002798).

### Data availability

The WGS data were generated at The Jackson Laboratory Genomics Core. Derived WGS data and all other cell line data supporting the findings of this study are available within the article and its supplementary data files or from the corresponding authors upon request.

## Results

### Depletion of ecDNA+ cell population after lentiviral transduction and selection for drug resistance

**Figure 1:**
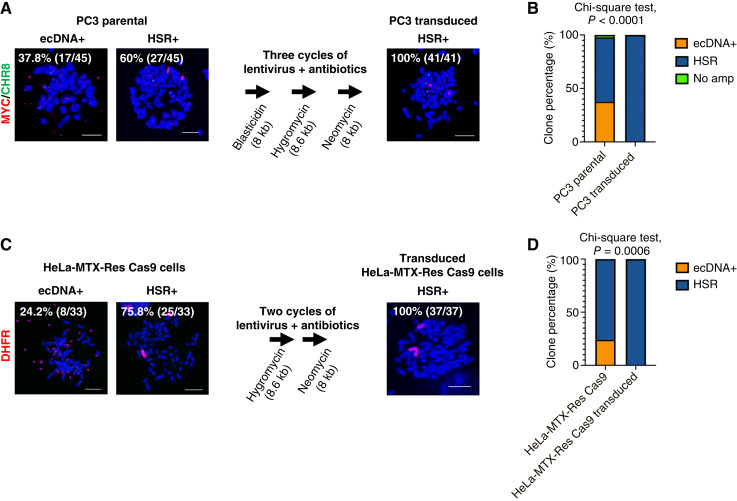
Lentiviral transduction causes the depletion of cancer cells with extrachromosomally amplified oncogenes. **A,** Oncogene amplification status before and after lentiviral transduction. Parental PC3 cells and the transduced PC3 cells that underwent three cycles of lentiviral transduction followed by antibiotics selection were synchronized at metaphase and processed for FISH analysis. MYC probe (red) and chromosomal control probe (green) were used. The number of cells containing *MYC* amplification was quantified (*n* = 41). Bar = 10 μm. The lentiviral genome size for each cycle is indicated, along with the name of the antibiotics used in that cycle. Cells were transduced with lentivirus for 48 hours and subjected to antibiotics selection for 1–2 weeks. **B,** Graphical summary and statistical test of FISH analysis (*χ*^2^ test, *P* < 0.0001). **C,** Oncogene amplification status before and after lentiviral transduction. The HeLa cell line that acquired methotrexate resistance (HeLa-MTX-Res) underwent two cycles of lentiviral transduction followed by antibiotics selection. Then, the cells were synchronized at metaphase and processed for FISH analysis. A DHFR probe (red) was used. The number of cells containing *DHFR* amplification was quantified (*n* = 33). Bar = 10 μm. The lentiviral genome size for each cycle is indicated, along with the names of the antibiotics used in that cycle. Cells were transduced with lentivirus for 48 hours and subjected to antibiotics selection for 1–2 weeks. **D,** Graphical summary and statistical test of FISH analysis (*χ*^2^ test, *P* = 0.0006).

**Figure 2:**
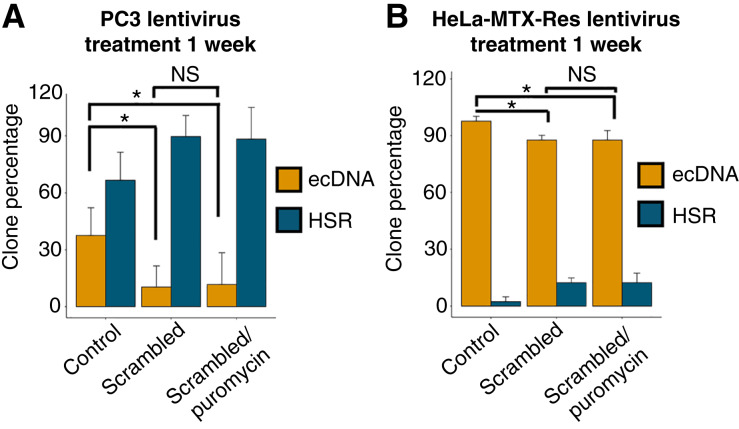
Antibiotic treatment does not cause depletion of cells with ecDNA. **A,** Parental PC3 cells (control), the lentivirally transduced PC3 cells (scrambled), and the PC3 cells that were lentivirally transduced for 48 hours and selected with puromycin for 1 week (scrambled/puromycin) were subjected to FISH analysis with the MYC probe. Cell subpopulations with ecDNA or HSR were quantified based on FISH images (*t* test, *, *P* < 0.05; NS = 3). We used the Invitrogen LentiArray CRISPR nontargeting control lentivirus particle for this experiment. **B,** HeLa-MTX-Res cells (control), the lentivirally transduced HeLa-MTX-Res cells (scrambled), and the HeLa-MTX-Res cells that were lentivirally transduced for 48 hours and selected with puromycin for 1 week (scrambled/puromycin) were subjected to FISH analysis with the DHFR probe. Cell subpopulations with ecDNA or HSR were quantified based on FISH images (*t* test, *, *P* < 0.05; NS = 3). We used the Invitrogen LentiArray CRISPR nontargeting control lentivirus particle for this experiment.

**Figure 3:**
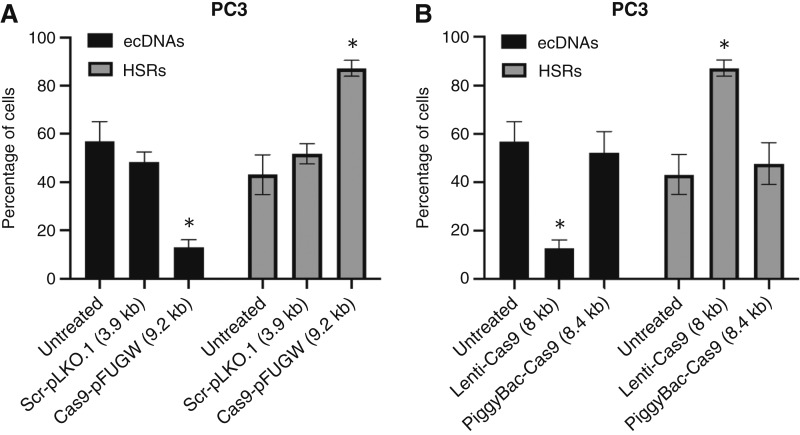
Alternative ways to deliver transgenes to cells without population shift. **A,** Larger transgene/plasmid size in lentiviral constructs depletes ecDNA+ cells with the expansion of HSR+ cells. PC3 cells were either untreated or lentivirally transduced with the indicated plasmids for 48 hours and selected with blasticidin for 2 weeks. FISH analysis with the MYC probe was performed to quantify subpopulations (ANOVA, *, *P* < 0.0001; *n* = 3). **B,** Non-lentiviral (PiggyBac) plasmid does not deplete ecDNA+ cells. PC3 cells were either untreated or lentivirally transduced with the Cas9 plasmid (Lenti-Cas9) or transfected with the PiggyBac-Cas9 plasmid for 48 hours and selected with blasticidin for 2 weeks. FISH analysis with the MYC probe was performed to quantify subpopulations (ANOVA, *, *P* < 0.0001; *n* = 3).

This phenomenon was first observed in PC3, a prostate cancer cell line model known to have extrachromosomal *MYC* amplification (13). We sought to integrate multiple DNA constructs into the genomes of PC3 cells using a lentiviral transduction system. After three cycles of lentiviral transduction and antibiotic treatment with blasticidin, hygromycin, and neomycin, we observed a depletion of cells containing *MYC* ecDNAs, whereas cells containing linear *MYC* amplification, referred to as HSR, seemed unaffected (Fig. 1A and B). We generated a methotrexate-resistant HeLa cell line (HeLa-MTX-Res) that develops ecDNAs containing DHFR. After the first lentiviral transduction with a Cas9-expressing plasmid and blasticidin treatment, we observed a significant increase in cells containing *DHFR* HSRs (75.8%; Fig. 1C and D). Two additional cycles of lentiviral transduction and antibiotic selection with hygromycin and neomycin led to a complete depletion of cells containing *DHFR* ecDNAs (Fig. 1C and D). Depletion of ecDNA+ cells was gradually carried out during the multiple cycles of transduction with lentivirus carrying different transgenes, suggesting that the content of viral particles has no role in this phenomenon (Supplementary Fig. S1). There are mainly two parts in the lentiviral transduction process: virus treatment and antibiotic selection. To evaluate what drives this phenomenon, we quantified ecDNA+ and HSR+ cell populations in lentivirally transduced cells with and without puromycin treatment, which is a major antibiotic selection method broadly used in CRISPR-based screening. We found that virus treatment alone resulted in the depletion of the ecDNA+ cell population, whereas antibiotic selection had no further effect on the reduction of ecDNA+ cell populations or the expansion of HSR+ cell populations in both PC3 (Fig. 2A) and HeLa-MTX-Res cell line models (Fig. 2B). It has previously been reported that the size of the inserted genome negatively correlates with lentivirus transduction efficiency (14). We evaluated the impact of genome size and found that smaller genomes (<4 kb) showed no effect on the depletion of the ecDNA+ populations (Fig. 3A). This observation indicates that the transduction efficiency of ecDNA+ cancer cells and non-ecDNA cancer cells is dependent on the size of the transgene and suggests that lentiviral delivery of small molecules such as single-guide RNAs and short hairpin RNAs can be achieved in ecDNA+ cancer cells.

The PiggyBac transposon system, which integrates transgenes into host genomes via transposases, did not affect the depletion of ecDNA+ cell populations (Fig. 3B). These results suggest that the lentiviral transduction method should be used with caution in ecDNA+ cells, especially those containing mixed subpopulations with different forms of oncogene amplification, as it alters the original ratio of cell subpopulations. Additionally, these results suggest the transposon system as an alternative way to deliver transgenes to ecDNA+ cells without perturbing the ratio of cell subpopulations.

### Possible causes of lentiviral transduction–mediated ecDNA+ cell depletion

**Figure 4:**
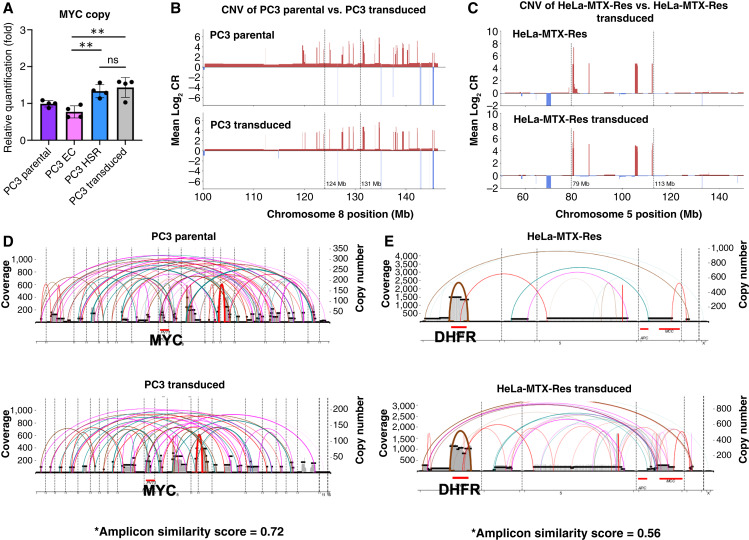
ecDNA reintegration does not cause the depletion of the ecDNA+ population. **A,** Relative quantification of MYC copy was tested by qPCR in parental, ecDNA+, HSR+, and lentivirally transduced PC3 cell lines (*t* test, **, *P* < 0.01; ns = 4). **B,** Copy-number variation (CNV) analysis results show a comparable copy-number ratio (CR) around the MYC amplification region between parental and transduced PC3 cells. **C,** CNV analysis results show a comparable CR around the DHFR amplification region between parental and transduced HeLa-MTX-Res cells. **D,** AmpliconArchitect results show a high similarity in the MYC amplicon structure between parental and transduced PC3 cells. *The closer it is to 1 in the range of 0–1, the more similar the structure is. **E,** AmpliconArchitect results show a high similarity in the DHFR amplicon structure between parental and transduced HeLa-MTX-Res cells. *The closer it is to 1 in the range of 0–1, the more similar the structure is.

Next, we sought to explain the relative depletion of ecDNA+ cells following lentiviral transduction. To directly compare the cell subpopulations with ecDNA or HSR, we performed single-cell cloning on PC3 cells. We identified two clones consisting of only cells containing extrachromosomal *MYC* amplification (PC3-ecMYC1 and PC3-ecMYC2) and two clones containing chromosomal *MYC* amplification (PC3-hsrMYC1 and PC3-hsrMYC2) using FISH analysis. We first sought to check whether the loss of ecDNA after lentiviral transduction occurred due to reintegration of ecDNA. We compared *MYC* copy numbers in parental, ecDNA+, HSR+, and transduced PC3 cell lines. The single cell–derived HSR+ PC3 clones showed comparable *MYC* copy numbers to the lentivirally transduced PC3 cells (Fig. 4A). Copy-number variation analysis results from WGS further confirmed that no copy-number changes occurred before and after lentiviral transduction (Fig. 4B and C). Structural analysis of oncogene amplifications (*MYC* and *DHFR*) using AmpliconArchitect showed highly similar amplicon structures before and after lentiviral transduction (Fig. 4D and E). In conjunction, the genome-wide DNA copy-number profiles showed near-identical similarity before and after transduction (Supplementary Fig. S2). These results collectively indicate that the depletion of the ecDNA+ cell population after lentiviral transduction is most likely due to the retention of the preexisting HSR+ subpopulation, rather than ecDNA reintegration.

**Figure 5:**
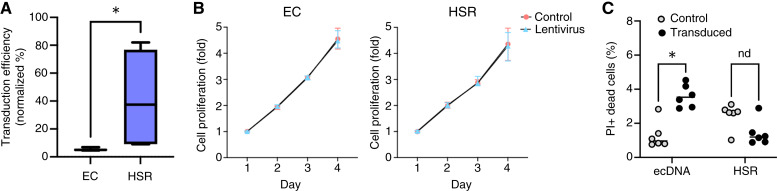
Low transduction efficiency and lethal effect of lentivirus in ecDNA+ cells lead to selective depletion. **A,** Differential lentiviral transduction efficiency in PC3 single-cell clones with MYC-ecDNA (EC) or MYC-HSR (HSR). Two single-cell clones per *MYC* amplification category were tested. Cells were transduced with lentivirus carrying green fluorescence protein (GFP, genome size = 6.4 kb) for 24 hours and subjected to flow cytometry analysis. Lentiviral transduction efficiency was calculated by quantifying the proportion of cells expressing GFP (*t* test, *, *P* < 0.05; ns = 3). **B,** Cell proliferation comparison between PC3 single-cell clones with EC or HSR. Two single-cell clones per *MYC* amplification category were tested. Five sets of cells were transduced with lentivirus carrying red fluorescence protein (mCherry, genome size = 4.5 kb), and each set of cells was subjected to the CellTiter-Glo assay on different days (1, 2, 3, 4, 5 days after transduction). The posttransduction proliferation rate was compared with the proliferation rate of the untreated control cells. **C,** Cell death analysis was performed in two isogenic pairs of PC3 clones. Cell death of each clone after 48 hours of lentivirus transduction (genome size = 4.5 kb) was analyzed by propidium iodide (PI) staining and compared with the nontransduced control cells (*t* test, *, *P* < 0.001; nd = 3 per clone).

Next, we sought to investigate whether ecDNA+ cell populations have reduced transduction efficiency compared with HSR+ cell populations. We evaluated lentiviral transduction efficiency on these four PC3 single-cell clones using a 9.6 kb lentivirus carrying a GFP-expressing plasmid. The single-cell clones were infected with lentivirus for 24 hours and subjected to flow cytometry analysis (Fig. 5A; Supplementary Fig. S3A). We observed that the ecDNA+ PC3 clones have a significantly lower transduction efficiency than those with the same oncogene on HSR (*t* test *P* value < 0.05). We confirmed the reduced transduction efficiency in ecDNA+ colorectal cells (COLO320DM) after 24-hour lentiviral infection compared with the isogenic pair of the same cells with HSR (COLO320HSR; *t* test, *P* value = 0.07; Supplementary Fig. S3B). Next, we evaluated transduction efficiency in a panel of three ecDNA+ and three HSR+ neuroblastoma cell lines, which consist exclusively of ecDNA+ or HSR+ cells. We observed the same result, with HSR+ neuroblastoma cell lines showing significantly higher transduction efficacy than ecDNA+ neuroblastoma cell lines after 48-hour lentiviral infection (two-way ANOVA, *P* value = 0.014; Supplementary Fig. S3C). These results collectively suggest that lentivirus infection may not be effective in the subset of cancer cells containing ecDNA. And a lower transduction efficiency drives the depletion of ecDNA+ cells following lentiviral transduction of a heterogeneous cell population and subsequent selection for drug resistance.

Relative depletion of ecDNA+ cells can also be achieved by a difference in the proliferation rate of ecDNA+ cells compared with HSR+ cells. Therefore, we investigated cell growth patterns at different time points of lentiviral treatment in both ecDNA+ and HSR+ PC3 clones (Fig. 5B; Supplementary Fig. S4A and S4B). The pure ecDNA+ or HSR+ PC3 clones were treated with lentivirus and tested for cell proliferation using the CellTiter-Glo assay. The ecDNA+ and HSR+ PC3 clones showed comparable cell proliferation patterns before and after lentiviral transduction, indicating that lentiviral transduction does not alter cell growth patterns.

Next, we analyzed cell death in response to lentiviral transduction in multiple cancer cell lines with ecDNA, HSR, or no gene amplification. Transduced ecDNA+ cell lines exhibited higher cell death compared with HSR+ and nonamplified cell lines (two-way ANOVA, *P* value = 0.0493), suggesting that lentiviral transduction can also result in selective lethality in ecDNA+ cells (Supplementary Fig. S4C). We then compared cell death events in the isogenic pairs of PC3 clones (two ecDNA+ clones and two HSR+ clones) before and after transduction. ecDNA+ PC3 clones showed significantly increased cell death after lentiviral transduction, whereas HSR+ clones showed no difference in cell death before and after transduction (Fig. 5C). Therefore, the selective depletion of ecDNA+ cells following lentiviral transduction seems to result from (i) their lower transduction efficiency, which can cause subsequent cell death of nontransduced cells by antibiotic treatment, and (ii) the selective lethal effects of the lentivirus.

**Figure 6:**
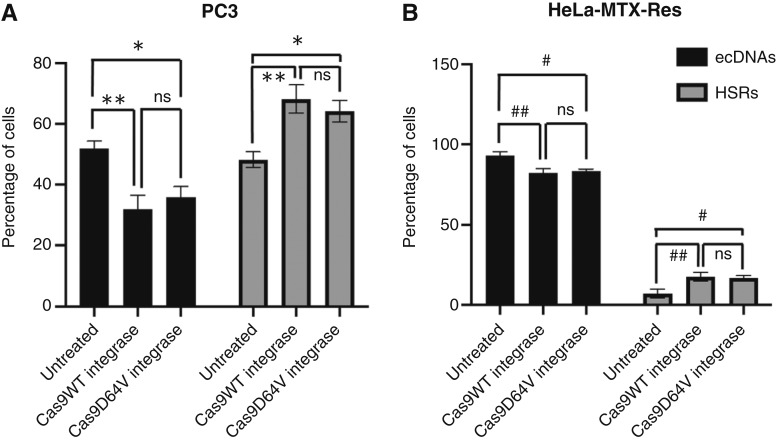
Integrase-deficient lentivirus does not rescue the effect of integrase-intact lentivirus on ecDNA depletion. **A,** PC3-NCI and (**B**) HeLa-MTX-Res cells were either untreated or transduced with Cas9-expressing lentivirus (genome size = 8 kb) generated either using wild-type integrase (Cas9 WT integrase) or point mutant integrase (Cas9 D64V integrase) that does not integrate into the genome. After 48 hours of transduction, cells were cultured for 2 weeks. FISH analysis with oncogene probes was performed to quantify subpopulations (ANOVA; *, *P* < 0.01; **, *P* < 0.05; #, *P* < 0.05; ##, *P* < 0.01; ns = 2).

We assessed the effect of integrase on the depletion of ecDNA+ cells in PC3 and HeLa-MTX-Res cell line models (Fig. 6). To achieve this, we used an integrase-defective lentiviral vector (D64V integrase), which mutation showed the strongest inhibition of integrase (15). Despite the lack of integrase functionality, lentiviral transduction depleted the ecDNA+ populations in both PC3 and HeLa-MTX-Res cell line models, suggesting the absence of a correlation between integrase function and ecDNA depletion.

Finally, we found that transducing PC3 cells using increased infection time (72 hours) resulted in an increase in GFP-expressing cells in ecDNA+ clones compared with HSR+ clones (Supplementary Fig. S5). Together with the comparable cell proliferation rate in ecDNA+ clones before and after lentivirus treatment, the increased transduction efficiency in ecDNA+ clones with longer exposure to lentivirus suggests that ecDNA+ cells may have delayed transgene expression compared with HSR+ cells. This observation provides an additional way to improve transduction efficiency in ecDNA+ cells. Future experiments are needed to better understand the difference between short- and long-term infection times.

## Discussion

In this study, we show that lentiviral transduction drives specific depletion of ecDNA+ cells from heterogeneous cell populations. This is mainly due to lower transduction efficiency in ecDNA+ cells when the lentiviral particles carry a large genome size. Our results indicate that ecDNA+ cancer cells may have an impaired reverse transcription process, as ecDNA+ cells were successfully transduced with a smaller genome (<4 kb) but not a larger size (>4 kb), which takes a longer time to complete reverse transcription. Lentiviral transduction is the most common method for the stable delivery of foreign genes and has, for example, been used in the very popular DepMap resource (16). Our results suggest that the use of lentivirus requires caution in ecDNA research. To ensure accurate interpretation of such data, thorough validation of ecDNA status will be necessary. This study suggests alternative methods to successfully achieve transgene delivery in ecDNA+ cell models. First, lentiviral delivery of plasmids with smaller genomes did not alter transduction efficiency. Prolonged infection time may result in an increased number of ecDNA+ infected cells. Second, the PiggyBac system did not affect subpopulation fraction shift, indicating that the transposon can be used alternately. Third, transgene delivery using the lentiviral system of a homogeneous ecDNA+ population, that is, in the absence of HSR+ and other subpopulations, may be successfully achieved by carefully tweaking to overcome differences in transduction efficiency and cell death following transduction.

Although our observations suggest a limited use for a common technology in ecDNA research, our results can also be interpreted in a positive way, as they may reveal a pathway with the potential to selectively deplete cells driven by ecDNA in cancer. Further molecular mechanism studies on the lower transduction efficiency at specific genome sizes and treatment times in ecDNA are essential as they will inform us about what makes ecDNA+ cells unique from other cancer cell subpopulations in the context of viral infection, which will lead to ecDNA-specific targeting strategies.

## Supplementary Material

Supplementary Figure S1 Figure S1. Gradual depletion of cancer cells with ecDNA.

Supplementary Figure S2 Figure S2. Whole-genome sequencing-derived DNA copy number profiles.

Supplementary Figure S3 Figure S3. Subpopulations with ecDNA tend to show lower lentiviral transduction efficiency.

Supplementary Figure S4 Figure S4. Comparison of cell proliferation between cancer single-cell clones with or without ecDNA.

Supplementary Figure S5 Figure S5. Subpopulations with ecDNA show modestly higher lentiviral transduction efficiency following long-term virus infection.
