# Supplementary figures and images for: Selective Depletion of Cancer Cells with Extrachromosomal DNA via Lentiviral Infection
Source: Cancer Res Commun. 2025 Aug 28;5(8):1458–65. doi: 10.1158/2767-9764.CRC-25-0144 (PMC12392265; doi:10.1158/2767-9764.CRC-25-0144)

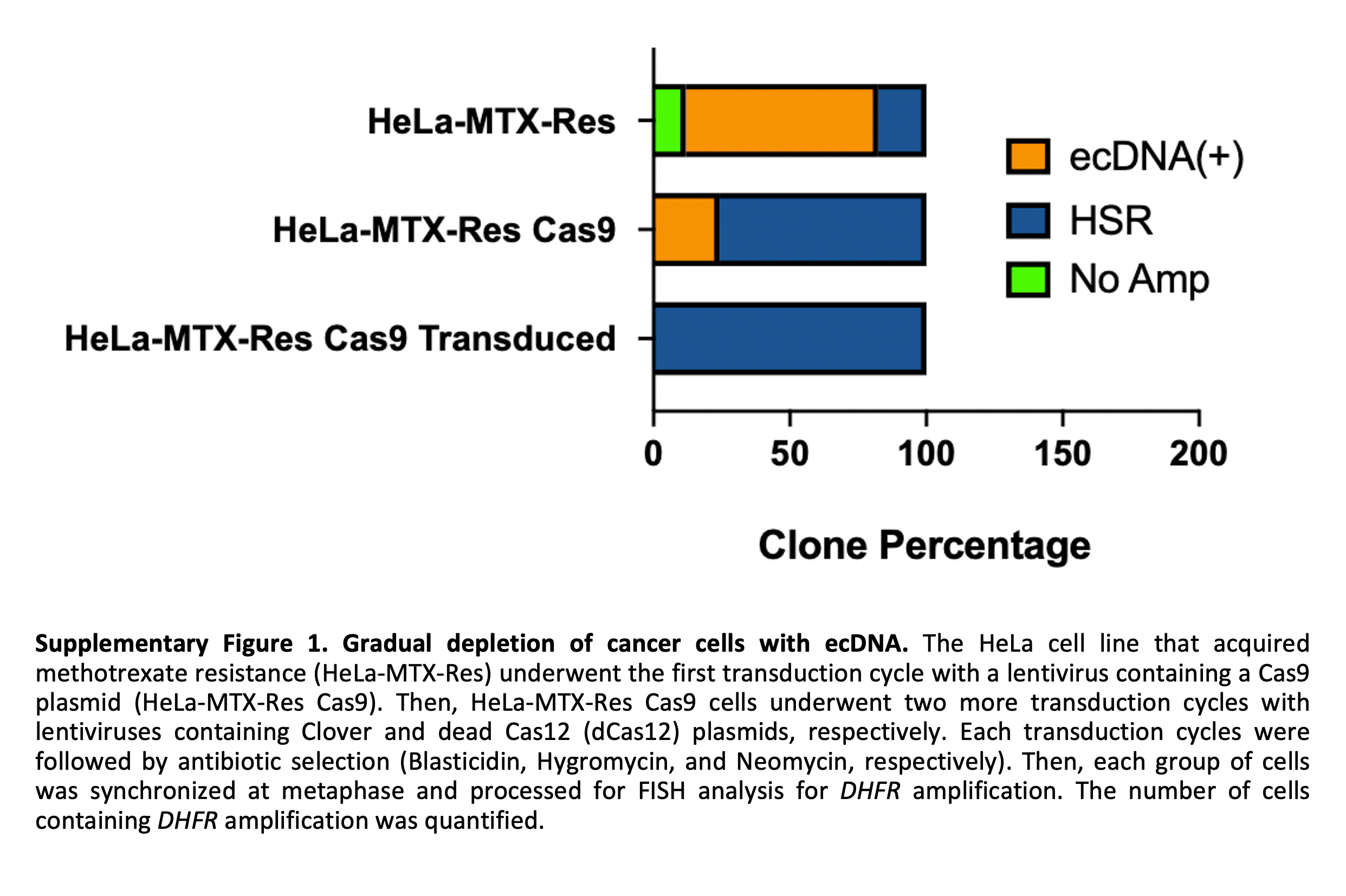

Supplement: Supplementary Figure S1 — Figure S1. Gradual depletion of cancer cells with ecDNA. [file crc-25-0144_supplementary_figure_s1_suppsf1.png]

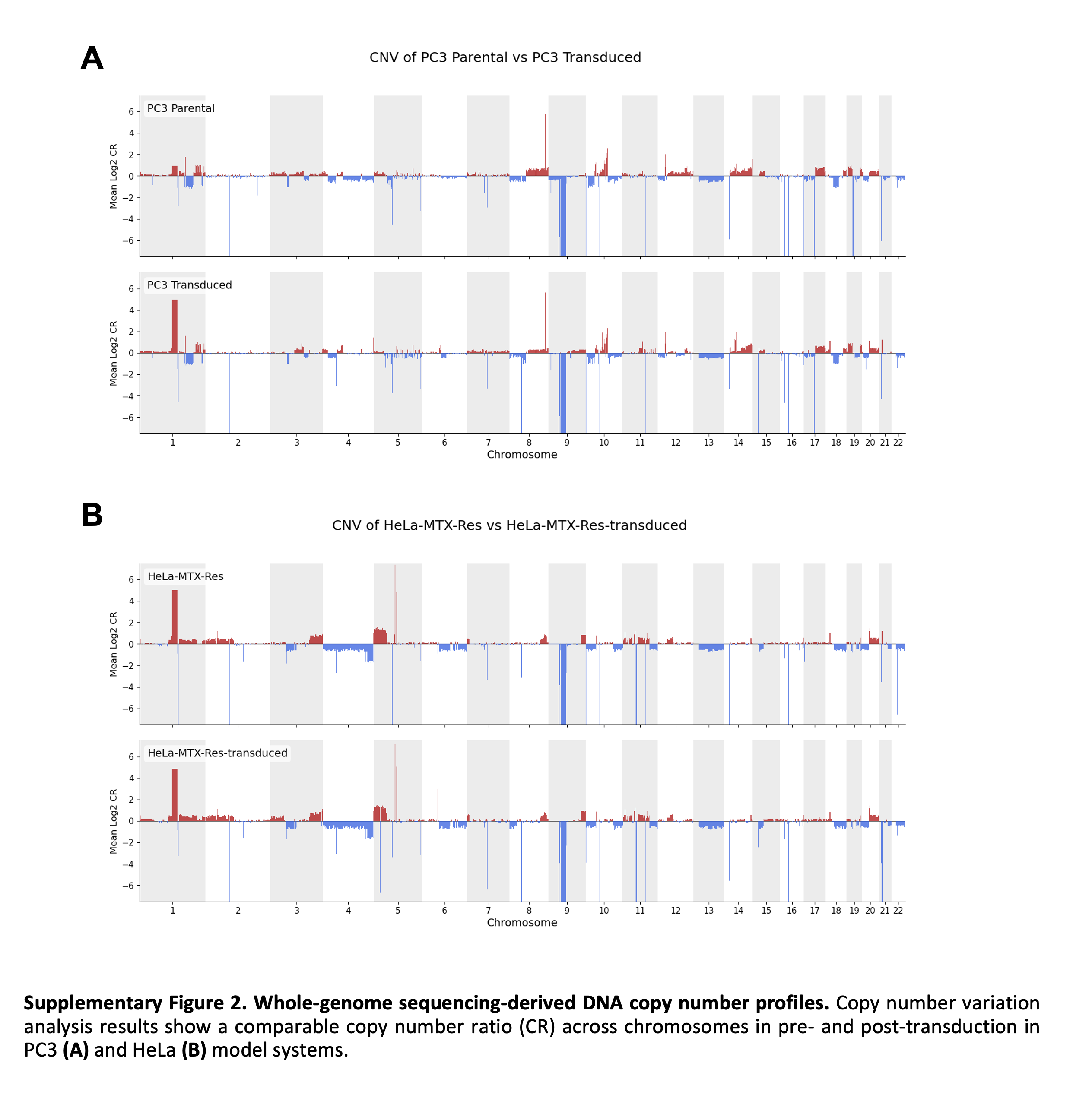

Supplement: Supplementary Figure S2 — Figure S2. Whole-genome sequencing-derived DNA copy number profiles. [file crc-25-0144_supplementary_figure_s2_suppsf2.png]

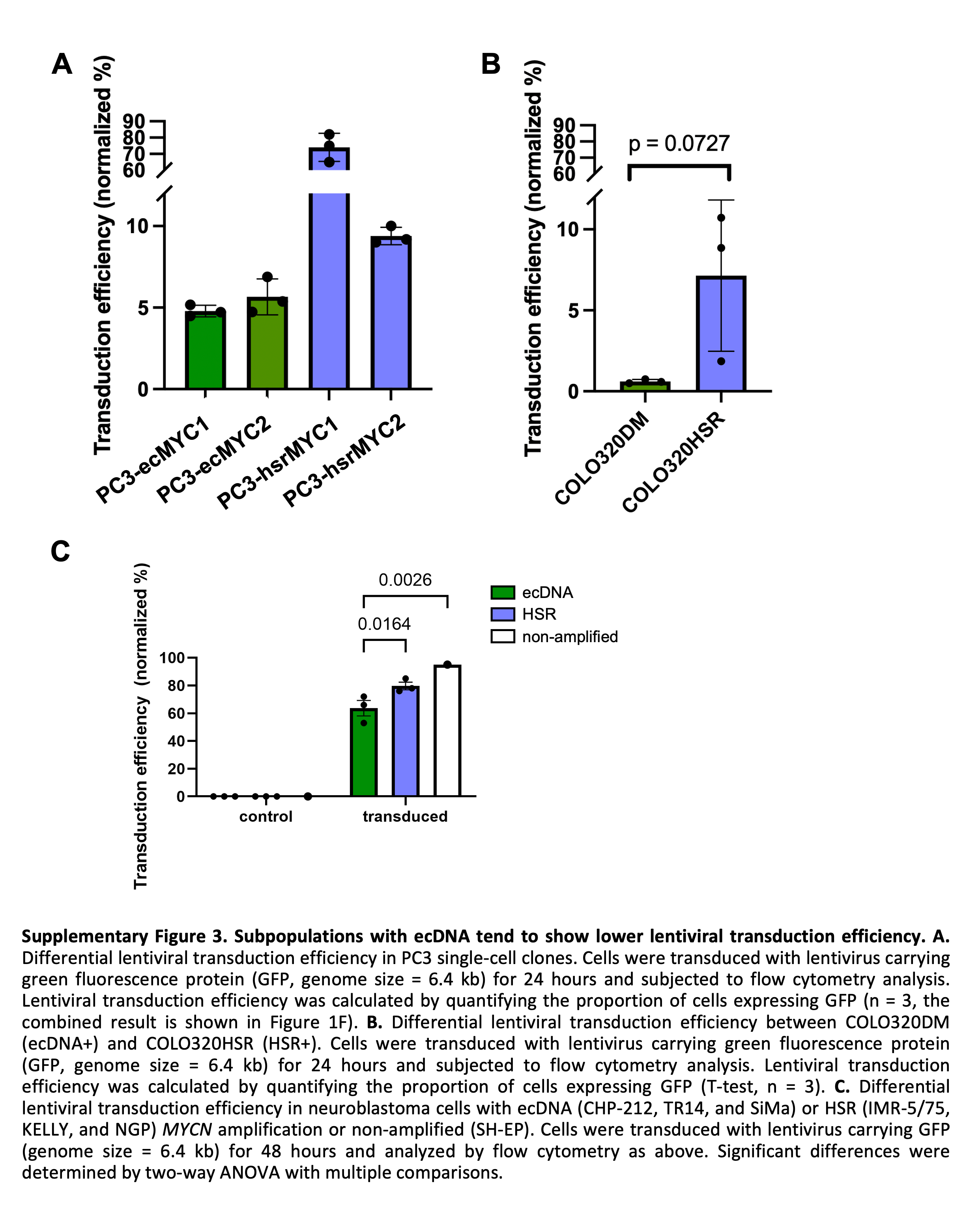

Supplement: Supplementary Figure S3 — Figure S3. Subpopulations with ecDNA tend to show lower lentiviral transduction efficiency. [file crc-25-0144_supplementary_figure_s3_suppsf3.png]

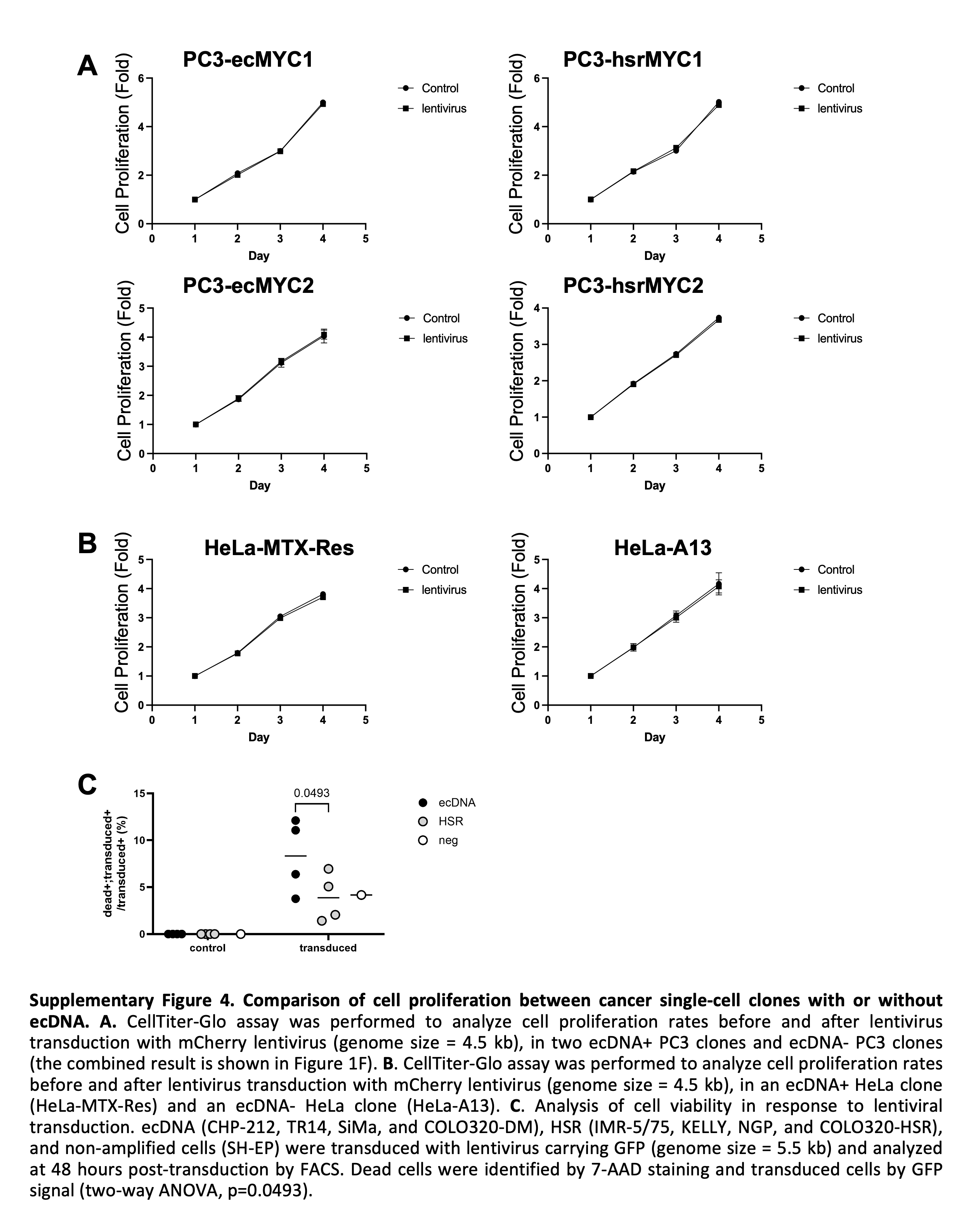

Supplement: Supplementary Figure S4 — Figure S4. Comparison of cell proliferation between cancer single-cell clones with or without ecDNA. [file crc-25-0144_supplementary_figure_s4_suppsf4.png]

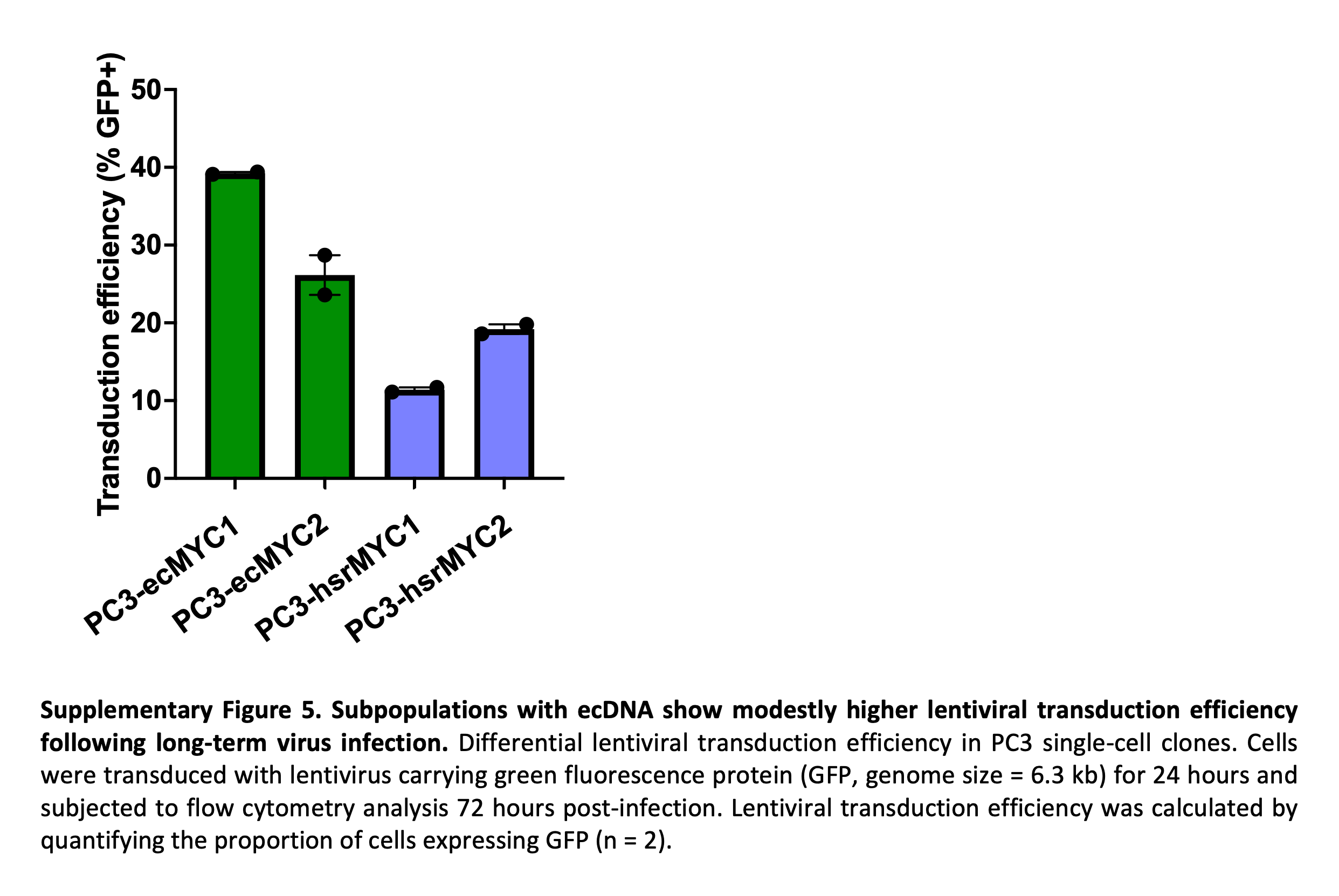

Supplement: Supplementary Figure S5 — Figure S5. Subpopulations with ecDNA show modestly higher lentiviral transduction efficiency following long-term virus infection. [file crc-25-0144_supplementary_figure_s5_suppsf5.png]
